# Pharmaceutical aerosols for the treatment and prevention of Tuberculosis

**DOI:** 10.3389/fcimb.2012.00118

**Published:** 2012-09-07

**Authors:** Shumaila N. M. Hanif, Lucila Garcia-Contreras

**Affiliations:** Department of Pharmaceutical Sciences, Collage of Pharmacy, University of Oklahoma Health Sciences CenterOklahoma City, OK, USA

**Keywords:** Tuberculosis, aerosols, inhaled vaccines, treatment

## Abstract

Historically, pharmaceutical aerosols have been employed for the treatment of obstructive airway diseases, such as asthma and chronic obstructive pulmonary disease, but in the past decades their use has been expanded to treat lung infections associated with cystic fibrosis and other respiratory diseases. Tuberculosis (TB) is acquired after inhalation of aerosol droplets containing the bacilli from the cough of infected individuals. Even though TB affects other organs, the lungs are the primary site of infection, which makes the pulmonary route an ideal alternative route to administer vaccines or drug treatments. Optimization of formulations and delivery systems for anti-TB vaccines and drugs, as well as the proper selection of the animal model to evaluate those is of paramount importance if novel vaccines or drug treatments are to be successful. Pharmaceutical aerosols for patient use are generated from metered dose inhalers, nebulizers, and dry powder inhalers (DPIs). In addition to the advantages of providing more efficient delivery of the drug, low cost, and portability, pharmaceutical dry powder aerosols are more stable than inhalable liquid dosage forms and do not require refrigeration. Methods to manufacture dry powders in respirable sizes include micronization, spray drying, and other proprietary technologies. Inhalable dry powders are characterized in terms of their drug content, particle size, and dispersibility to ensure deposition in the appropriate lung region and effective aerosolization from the device. These methods will be illustrated as they were applied for the manufacture and characterization of powders containing anti-tubercular agents and vaccines for pulmonary administration. The influence of formulation, selection of animal model, method of aerosol generation, and administration on the efficacy demonstrated in a given study will be illustrated by the evaluation of pharmaceutical aerosols of anti-TB drugs and vaccines in guinea pigs by our group.

## Introduction

Despite being treatable and, indeed, preventable, tuberculosis (TB) continues to be a major public health challenge in many parts of the world (Fair et al., 2007). The number of people infected with TB is growing as reflected by world-wide increases in new cases at an incidence rate of 1.8% per year between 1997 and 2000 (Corbett et al., 2003). In 1993, the number of people infected with TB in the world was so large that the World Health Organization (WHO) declared it “a global emergency” (Expert Committee, 2006). Recently, a survey by the WHO estimated that one third of the world population (≈2 billion) is infected with tubercle bacilli, 8 million people develop active disease and 2 million die of TB each year (Barnes and Cave, 2003; Frieden et al., 2003).

In 1995, the WHO recommended the implementation of a treatment strategy called Directly Observed Therapy, Short-course (DOTS). The aim was to treat TB infected patients by directly observing them to take their medication (isoniazid, rifampicin, pyrazinamide, and streptomycin or ethambutol or both) for at least the first two months of treatment. This ensured that the medication was taken in the right combinations and appropriate dosage, which has reduced the incidence of multi-drug resistant TB. It is anticipated that 80% of deaths attributed to TB world-wide will be prevented by using DOTS. The estimated global incidence rate of TB peaked around 2003, but after the successful introduction of DOTS it decreased between 2005 and 2006 (WHO, 2008). In 2006, the incidence rate stabilized in the European Region and continued to decline slowly in the other five WHO regions of the world. The WHO estimates that the prevalence and death rates have been falling longer and faster than the incidence rate.

A person is infected with TB shortly after the inhalation of coughed/sneezed droplets containing as few as 3–5 *Mycobacterium tuberculosis* bacilli from an already infected person (Dannenberg, 1999). The first host defense is the alveolar macrophages that scavenge the alveolar surface ingesting the bacilli and becoming non-specifically activated during the process. If the host is immunocompetent, the bactericidal capacity of the macrophage will destroy the bacteria or limit its multiplication, but if the host is immune-compromised or has a weak immune response, *M. tuberculosis* bacilli (MTB) is able to counteract the host's defenses. MTB can impair innate macrophage anti-microbial mechanisms by inhibiting the phagosome-lysosome fusion (Nardell, 1993). If the macrophage is able to defeat the host defense, MTB can replicate within the macrophage causing it to rupture. Thus, the main weapons of MTB against the human host are its ability to multiply logarithmically within non-activated macrophages and extracellularly in liquefied caseous material, including those proximal to the lumen of cavities. For these reasons, this bacterium is considered to be among the most successful and poorly understood pathogens affecting human beings.

After the rupture of infected macrophages, immature macrophages (monocytes) are attracted from systemic circulation by chemotactic factors initiating the granuloma formation (Nardell, 1993). Macrophages are also responsible to process the antigens of MTB and present them to the specific T lymphocytes, although recent theories suggest that dendritic cells and alveolar macrophages may share this role (Kaufmann, 2004). Likewise the current dogma is that cell mediated immunity (CMI) is the only defense of the host to MTB, but others had suggested that mucosal immunity may also be important (Dietrich et al., 2006). Even before the CMI is mounted completely in the host, MTB is able to escape the lung environment and enter the bloodstream to spread to other organs, notably the spleen and liver. All of these events can occur even before is noted that a patient has been infected.

The tuberculin skin test has been used as a method to diagnose TB in epidemiological studies in humans and cattle for more than half a century (De Souza, 2009). It consists of an intradermal injection of a purified protein derivative (PPD), a poorly defined mixture of *M. tuberculosis* antigens containing both secreted and somatic proteins. Some of the mycobacterial antigens contained in PPD are shared among pathogenic mycobacteria belonging to the *M. tuberculosis* complex (*M. tuberculosis*, *M. bovis*, and *M. africanum*), environmental non-tuberculous mycobacteria (NTM), and the vaccine substrain *M. bovis* bacille Calmette-Guerin (BCG) (Van Pinxteren et al., 2000). Thus, although responses to PPD are an important aid in the diagnosis of TB and can give an indication of exposure to mycobacteria, it is often impossible to distinguish BCG vaccination and exposure to NTM from *M. tuberculosis* infection. Therefore, it has been apparent that a new diagnostic reagent with specificity for *M. tuberculosis* and *M. bovis* is needed to overcome the limitations of PPD (Hanif et al., 2010b).

BCG vaccination is considered the most important tool to protect against TB. According to the WHO, more people alive today have been vaccinated with the live attenuated BCG vaccine than with any other vaccine. In spite of its widespread use and many advantages like being inexpensive, safe at birth, given as a single shot, and provision of some protection against leprosy, BCG vaccination remains controversial. Its protective efficacy has varied widely in different parts of the world and its impact on the global problem of TB remains unclear (Rodrigues and Smith, 1990; Haile and Kallenius, 2005). Estimates of protection imparted by BCG against pulmonary TB vary greatly from 0 to 80% (Castanon-Arreola and Lopez-Vidal, 2004). A trial in British school children, in 1952, showed 77–84% efficacy (Rodrigues and Smith, 1990), where as the Chingleput trial in India showed zero efficacy of protection provided by the BCG vaccine (Tripathy, 1987). This variability has been attributed to various factors including strain variation in BCG preparations, environmental influences such as sunlight exposure, poor cold-chain maintenance, genetic or nutritional differences between populations and exposure to environmental mycobacterial infections (Bloom and Fine, 1994; Fine, 1995; Haile and Kallenius, 2005).

Implementation of BCG vaccination faces two other problems; BCG vaccination induces a delayed type hypersensitivity (DTH) response that cannot be distinguished from exposure to *M. tuberculosis* as BCG and PPD are sharing most of the antigens, making the use of tuberculin skin test unreliable for diagnostic or epidemiological purposes (Lowrie et al., 1995; Mustafa and Al-Attiyah, 2003; Teixeira et al., 2006). Second, it cannot be used indistinctly in all populations, as the WHO has recommended that children with symptoms of HIV infection should receive all the vaccines except BCG (Hesseling et al., 2003; Pardini et al., 2006). The reason is that BCG is a live attenuated vaccine that may cause disease in immune-compromised people rather than giving immunity. Thus, there is an urgent need to develop more specific and safer vaccines against MTB. Development of a vaccine that would provide better protection than BCG (either whole organism or subunit vaccines) requires the identification of the immunological mechanisms that lead to protection against infection and of the antigens recognized by those protective responses (Hanif et al., 2010a).

Treatment of TB infection is an arduous and lengthy process requiring daily co-administration of 2–4 antibiotics for at least six months (Fox et al., 1999). For most patients, regimens that contain the first line drugs rifampicin and isoniazid can be completed in six months if pyrazinamide is included and in nine months, if pyrazinamide is omitted. However, treatment for bacterial strains resistant to isoniazid or rifampicin that require the use of second line drugs such as capreomycin, amikacin or kanamycin are continued for minimum of 12 months for up to 24 months. The extended use of these antibiotics frequently produces unwanted side effects in patients due to intrinsic drug toxicity. During this extended therapy, patients may terminate treatment early due to many factors like unwanted side effects or alleviation of primary symptoms (Thomas, 2002). Partial treatment can make the disease more difficult to treat since selection of drug resistant mutants of *M. tuberculosis* may occur (Garcia-Contreras et al., 2010).

The world-wide increase in the incidence of TB, inadequate, and non-specific immunological methods of diagnosis, the failure of BCG vaccine to protect against TB in all populations and the lengthy treatment duration have spurred the need for improved diagnostic tests, new vaccines, and new therapies against a disease. Thus, successful strategy to control TB on a global scale requires: (1) improved methods for early diagnosis of an infection; (2) an effective vaccine that can be given safely to all populations and in all parts of the world to protect against all forms of the disease; and (3) advances in TB therapy that reduces frequency of dosing and over all treatment time and side effects, that can be effective against MDR/XDR and safe for HIV infected TB patients (Mustafa, 2009; Garcia-Contreras et al., 2010). Recent advances in these areas will be covered in the following sections.

## Animal models

Animal models are extremely important in any type of research as they allow manipulation of different variables involved that may not be easy or feasible to do in human subjects. Initial studies in virtually every area of pharmaceutical and pharmacological research have been performed in small rodents because terminal procedures can easily be conducted and large number of these animals can be used for statistical validity (Cryan et al., 2007). Rats and guinea pigs are frequently used for the study of drug delivery to lungs because a variety of dosing techniques that requires a small amount of drug can be employed. However, to study novel vaccines (not inhaled) and treatments for TB, a large number of studies have used mice and fewer have used other animal models including guinea pigs, rabbits, monkeys, and non-human primates. Among the advantages that make the mouse the animal model most used in TB studies are the vast knowledge of its immune system, the large variety of reagents available to study their immune response, their cost, and the number of knock out species available (Orme, 2003). In addition to the differences of these species and humans in the progression of the disease, the tidal volume and breathing frequency of mice are significantly different from humans (Hickey and Garcia-Contreras, 2001; Cryan et al., 2007), which makes delivery of large doses of aerosol to mice is difficult to achieve in short periods of time and amount of biological fluids that can be collected is small. This, plus the fact that all rodents are obligate nose breathers makes this animal model less than appropriate to study novel inhaled vaccines or treatments for TB. Although rats can be comparable to humans for the study of diseases such as influenza (Huang et al., 2003) and pulmonary fibrosis (Spond et al., 2003), they are not very susceptible to TB infection, neither by the IV route nor by aerosol exposure (Ornstein and Steinbach, 1925).

The comparative biology of the guinea pig indicates that the physiology of their pulmonary tract is quite similar to that of humans; particularly the response of the lung to inflammatory stimuli as well as the dermal response to both acute and chronic inflammatory mediators (Wagner and Manning, 1976; Lechner and Banchero, 1982). Hormonally and immunologically guinea pigs are more like human than other rodents, thus they are often used to model human infectious disease (McMurray, 1994). Guinea pigs are susceptible to aerosol infection with only a few MTB and the progression of the disease (bacillemic phase and formation of granulomas) is similar to that in humans (McMurray, 2003). Guinea pigs are also considered the “gold standard” to test the potency and standardize PPD for human use, since they are the smallest species that can develop indurate delayed hypersensitivity reaction in the skin after injection of this reagent and the procedure can be performed without sacrificing the animal (McMurray, 2001). Besides the limitation in the number of immunological reagents available to study responses in this animal model, they share with the other rodents the differences in pulmonary function and respiratory parameters with humans. This makes the administration of daily aerosol doses of anti-tubercular drugs very difficult to match with the true inhaled dose that can be achieved in a human. Thus, the selection of the appropriate dosing device and the selection of a formulation optimized for pulmonary delivery are critical for the success of this approach. These factors will be discussed in subsequent sections. Nevertheless, guinea pigs are an excellent model to test inhaled vaccines that require on 1–3 administrations.

Merely from the delivery point of view, the easiness of aerosol delivery to conscious animals and the amount of aerosol delivered increase as the size of the animal increases. Rabbits, non-human primates, and calves are larger animal models that have been employed to study different aspects of TB infection and treatment. Each of them has its particular advantages: rabbits is the smallest animal model to study cavitary TB (Dharmadhikari and Nardell, 2008), non-human primates are great models to study co-infection of TB with HIV that can be applicable to humans (Mattila et al., 2011), and calves are frequently used to develop diagnostic reagents to differentiate between vaccinated and infected individuals because large volumes of blood can be drawn without sacrificing the animal (Pollock and Andersen, 1997). However, there are no studies reported in the literature that use aerosol delivery of vaccines or drugs in these larger animal models for the treatment or prevention of TB. Perhaps a reason for this is the high cost associated with the purchase and maintenance of these animals as well as ethical issues. It is conceivable that after the efficacy of an approach is tested in the smaller animal models, a logical step would be to evaluate such approach in a larger animal model, in which larger amounts of a therapeutic aerosol may be delivered.

## Methods of aerosol delivery for anti-tubercular drugs and vaccines

Even though metered dose inhalers are the devices most commonly used in inhaled therapies for humans, they are limited by the size of the dose they can deliver with one puff. These small dose sizes are suitable for potent therapeutics such as those used in the treatment of asthma, but they are significantly smaller than the doses required to deliver antibiotics for the treatment of a disease such as TB. Thus, it is not surprising that there are no reports of their use in TB treatment or vaccines.

Perhaps the first documented use of inhaled therapy for TB treatment in humans is the work of Paraf et al. in 1953 using liquid aerosols of antibiotics (Paraf et al., 1953), whereas the first use of an inhaled vaccine against TB was reported by Rosenthal et al. (1968). Historically, most antibiotics and experimental vaccines that have been delivered by the pulmonary route have been delivered from liquid formulations, and the aerosols have been generated by nebulizers. Except for the Pari eflow, the Activaero AKITA and the APIXNEB systems, most nebulizers are inefficient, delivering only about 10% of the initial vaccine/drug load to the patient (Garcia-Contreras, 2012). This efficiency is significantly decreased when nebulization is used to deliver aerosol to conscious rodents, because their much smaller lung capacity and breathing frequency, and the fact that they only breathe through their nose as explained in the previous section. Moreover, the use of nebulizers to deliver vaccines has been reported to decrease the potency of vaccines because of the shear force produced by the nebulizer to generate the aerosol (Coates et al., 2006).

For animal studies, the limitations on the delivery of solutions have been addressed by the use of direct administration methods. The main advantages of direct administration are that small and relatively large doses of drug can be delivered by these methods and that the dose delivered can be accurately measured. The main disadvantage is that the use of these methods for multiple or consecutive dosing may not be recommended, since they are performed under anesthesia or light sedation and the insertion of the device may cause local irritation. Direct administration methods for liquids include liquid instillation and spray instillation but only the latter delivers drug or vaccine in a form proxy to true aerosol. In general, they involve visualization of the trachea of the animal with the help of a laryngoscope to place a thin stainless steel tube in the trachea, near the carina, to administer drugs (Cryan et al., 2007). Liquid instillation, usually performed with oral gavage needles, delivers drugs in the form of very large and coarse droplets. The main disadvantage of this method is that the liquid may be distributed mainly in the larger airways and an unknown amount of the drug may be coughed up or swallowed (Hickey and Garcia-Contreras, 2001). Depending on the volume delivered, it also may cause significant stress in the subject or have the risk of endangering the animal. These limitations can be addressed by delivering solutions in smaller droplets produced by a sprayer which is denominated “spray instillation.” The MicroSprayer™ (Penn Century, Philadelphia, PA) is an example of such devices in which the aerosol is generated. The aerosol is generated by the atomizer in the tip of a long, thin, stainless steel tube of the MicroSprayer™ to deliver the liquid vaccine or drug. The size of the aerosol generated is influenced by the physo-chemical characteristics (density, viscosity, concentration) of the solutions/suspension to be delivered (García-Contreras and Hickey, 2003).

**Figure 1:**
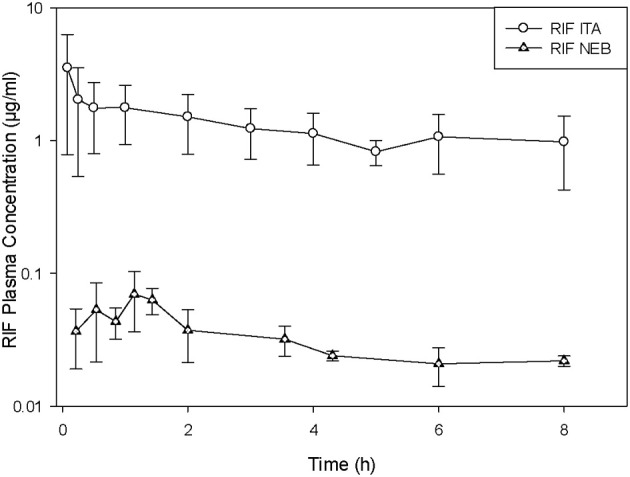
**Plasma concentration versus time curves in guinea pigs after administration of rifampicin suspension (RIF) by intratracheal liquid instillation (ITA) and nebulization (NEB).** (Average ± standard deviation, *n* = 3–5). Modified from García-Contreras and Hickey (2002).

Delivery of vaccines by direct administration methods is very efficient and ensures that the entire dose is delivered to the lung. For drugs, the use of direct administration methods is of paramount importance to determine the pharmacokinetic parameters of disposition after pulmonary delivery. The dose can be accurately measured and delivered by direct administration, but as explained earlier, when passive inhalation only a fraction of the nominal (loading) dose will be actually deposited in the lungs. Figure 1 illustrates this concept by comparing the plasma concentrations obtained after administration of the same nominal dose of rifampicin by intratracheal instillation and by nebulization (García-Contreras and Hickey, 2002).

Despite of all their advantages, direct administration methods may not be feasible for TB treatment since daily doses of the antibiotics are required for an extended period of time (weeks, in most cases). In addition to the risk of anesthetizing/sedating the animal multiple times, the repeated insertion of the tube may cause tracheal irritation and exacerbate the disease. Therefore, for studies of this nature, passive inhalation methods are preferred. In general, the conscious animals are placed in exposure chambers that can be for whole-body, head-only, and nose-only (Dorato, 1990). Animals can be placed in whole body chambers without restrain, but it is possible that the aerosolized drug also may be absorbed by the oral and percutaneous routes. In the first novel approach of its kind, a custom made whole body exposure chamber was employed to treat TB-infected guinea pigs with aerosols generated by an Acorn nebulizer from rifampicin suspensions (Suarez et al., 2001a,b). A one-log reduction in the bacterial burden of the lungs of these animals was reported after this treatment with rifampicin aerosols. Commercial whole body exposure chambers include those manufactured by CH technologies Inc. (Westwood, NJ) and EMMS (Hants, UK).

Head-only and nose-only chambers are used when aerosol exposure to other body parts needs to be restricted. These chambers consist of a cubic or cylindrical reservoir with cylindrical animal holders in its perimeter. The animal holders have a conical shape with the narrowest part designed to fit the animal's nose. The aerosol is circulated in the main reservoir or in a central duct and is distributed to all the animal holders. Commercially available nose only chambers have the capacity to hold 4–48 animals at one time for aerosol exposure, including those manufactured by CH Technologies (Westwood, NJ), Intox Products (Edgewood, NM), TSE GmbH (Badhamburg, Germany), and ADG Developments (Herts, UK). Providing that the mass flow (dose rate) and particle size and distribution remain constant during the aerosolization process, each animal will be exposed to the same aerosol dose. The dose can be calculated as recommended by the Association of Inhalation Toxicologists (AIT) from the aerosol concentration, the respiratory minute volume of the animal and its weight, the duration of exposure, and the respirable fraction of the aerosol (Alexander et al., 2008). The exposure chamber manufactured by ADG Developments is designed to deliver aerosols to approximately twenty rodents (Garcia-Contreras, 2011) and was employed to deliver aerosols generated by the Acorn nebulizer from rifampicin suspensions to TB infected animals daily for four weeks (Garcia-Contreras et al., 2006c). As a result of this treatment, a decrease was observed in the bacterial burden of the spleens of these animals compared to untreated controls.

An additional limitation of using solutions to deliver therapeutics (vaccines or drugs) against TB is the formulation of the therapeutic compound by itself. The properties and characteristics of the formulation as solution or suspension are limited by the solubility, stability, and sterility of drugs or vaccines in solution, as well as their requirements for storage. This is further complicated if the compound is completely insoluble or is formulated into engineered particles, because it may be difficult to find a biologically friendly media to suspend particles before nebulization and the concentration that can be achieved in the suspension may not be therapeutic for a given drug. Nebulization of highly concentrated suspensions may also result in particle precipitation in the nebulizer and the delivery of dilute aerosols (Smyth et al., 2003). All these limitations observed with solutions and suspensions have prompted researchers to find alternative formulations and strategies to deliver inhaled therapeutics. Dry powder formulations are considered the most stable from all inhaled dosage forms and can be delivered by dry powder inhalers (DPIs). Their popularity is such that several drugs previously formulated into metered dose inhalers are now manufactured into DPIs. Advantages of DPIs over nebulizers and metered dose inhalers include: portability, low cost, and better control of the dose delivered. DPIs can be classified into single-dose or multi-dose devices, depending on the number of doses that can dispense, or into passive or active, depending on the mechanism of powder dispersion (Hickey and Crowder, 2007). The efficiency of delivery for passive DPIs is influenced by its mechanism of dispersion and the inspiratory pressure of the patient but it ranges from 12 to 40%. The efficiency of delivery is higher for active (battery-driven) DPIs since the powder dispersion does not depend on the inspiratory pressure of the patient.

For animal studies, the Dry Powder Insufflator™, a unique device patented by (Philadelphia, PA) is used to deliver therapeutic powders by direct administration (Cryan et al., 2007). The main body of the insufflator has a small chamber that is filled manually with a small amount of pre-weighed dry powder. The powder is then dispersed in the chamber of the insufflator by applying small “puffs” of air with an empty plastic syringe. The insufflator can be used to deliver powders of different sizes, from nano-particles (Tetsuya and Hiroaki, 2006) to large porous particles (Codrons et al., 2004). The particle size distribution of the insufflated powders are essentially unaffected by passage through the device, as measured by a variety of sizing techniques (Codrons et al., 2004; Tetsuya and Hiroaki, 2006; Lu et al., 2007). Our group has employed this device to study the disposition of a number of anti-tubercular compounds when delivered by the pulmonary route to guinea pigs, including rifampicin (García-Contreras et al., 2006a; Sung et al., 2009b), capreomycin (Garcia-Contreras et al., 2007, 2012; Fiegel et al., 2008), and PA-824 (Sung et al., 2009a). For multiple administration of aerosolized powders by passive inhalation to multiple rodents, Hickey et al. designed the dry powder dispersion chamber (Garcia-Contreras et al., 2007). The design is similar to that of the nose-only exposure chamber but the volume of the reservoir is smaller and the powder is dispersed without the need of external air to achieve a highly concentrated standing cloud of the therapeutic powder. This novel chamber has been used to deliver daily doses of capreomycin (Garcia-Contreras et al., 2007) and PA-824 (Garcia-Contreras et al., 2010) for four weeks to treat TB-infected guinea pigs. The results of these studies will de discussed in subsequent sections.

## Formulation of anti-tubercular drugs and vaccines for inhalation

The preparation of novel inhaled therapeutics into dry powders is the most viable approach to produce stable dosage forms because there are a number of ways to manufacture them and scale up their production if needed. As mentioned before, powder formulations have greater stability than liquid formulation and usually do not require refrigeration. They are typically packed in such a way that protects them from light and humidity, which enhances their stability. Dry powders for inhalation can be prepared by micronization of the drug, or formulation into particles of different sizes and compositions, which also allows the control of their release from such systems. Micronized antibiotics have to be prepared into a blend with a carrier, usually lactose, in order to be dispersed, and efficiently inhaled. Micronized gentamicin powders have been delivered by the pulmonary route for the treatment of infections associated with cystic fibrosis (Goldman et al., 1990), but there are no reports of the use of micronized antibiotics for TB treatment. There are a considerable number of reports in the literature formulating anti-tubercular drugs and vaccines into microparticles and nanoparticles of different compositions for pulmonary delivery. For drugs, these formulations most commonly employ first line agents such as rifampicin, isoniazid, and pyrazinamide, but other second and third line drugs have also been investigated (Table 1). Particle cores are made of biodegradable polymers, lipids, sugars or aminoacids, and microparticles are more employed than nanoparticles.

**Table 1:** **Novel inhalable formulations for anti-tubercular drugs**.

**Drug**	**Formulation**	**Results**	**References**
Rifampicin	Poly(lactide-co-glycolide) microspheres	Single and double doses resulted in significantly reduced numbers of viable bacteria and lung damage	O'Hara and Hickey, 2000; Suarez et al., 2001a,b
–	Poly(lactide-co-glycolide) microspheres	Drug release accelerated by adsorption of pulmonary surfactant onto particles	Tomoda and Makino, 2007
–	Poly(lactic acid) microparticles	Optimization of parameters of manufacture	Patomchaiviwat et al., 2008
–	poly(lactic-co-glycolic acid) nanoparticle-containing mannitol microspheres	One-step preparation of microspheres; effective uptake by rat alveolar macrophages	Ohashi et al., 2009
Isoniazid and Rifampicin	Poly(D,L-Lactic Acid) microparticles	Particles taken up by cultured macrophages and intracellular drug concentrations higher than with drug alone	Sharma et al., 2001
Rifampicin, isoniazid, and pyrazinamide	Poly(DL-lactide-co-glycolide) nanoparticles	A single dose resulted in therapeutic plasma levels for 6–8 days and in the lungs for up to 11 days	Pandey et al., 2003
–	Solid lipid nanoparticles made of stearic acid	A single dose resulted in therapeutic plasma levels for 5 days and in lungs, liver, and spleen for 7 days	Pandey and Khuller, 2005
–	Nanoparticles made of sodium alginate	A single dose resulted in lung, liver, and spleen concentrations above MIC for 15 days	Zahoor et al., 2005
Isoniazid and rifabutin	Poly(lactic acid) microparticles	High drug payload released over 10 days; drug concentration in macrophages 20-fold larger than with solutions	Muttil et al., 2007
Para-aminosalicylic acid (PAS)	Large porous particles made of DPPC	PAS remains at therapeutic concentrations in the lung tissue for at least 3 h after insufflation	Tsapis et al., 2003
Capreomycin sulfate	Poly(lactide-co-glycolide) microspheres	Large porous microspheres in respirable sizes prepared by a simple method	Giovagnoli et al., 2007
–	Porous particles made of leucine and DPPC	Good aerosolization and stability properties. Longer half-life in guinea pigs	Fiegel et al., 2008
–	–	Significantly smaller lung bacterial burden, inflammation, and histopathological damage than IM controls	Garcia-Contreras et al., 2007
PA-824	Porous particles made of leucine and DPPC	Easily dispersable particles. Half life 2-fold longer after pulmonary administration than by oral gavage. Drug present in lung even after plasma levels were undetectable.	Sung et al., 2009a
–	–	Animals treated with inhaled particles had smaller degree of inflammation and histopathology damage than those receiving oral treatment.	Garcia-Contreras et al., 2010

Most of these particles were manufactured essentially by emulsification/solvent evaporation or spray drying, or a variation of these. The emulsification/solvent evaporation is a simple method that involves emulsification of a dispersed phase in which the core of the particle and the drug have been dissolved or dispersed into a continuous phase in which neither the drug or core material are soluble (Watts et al., 1990). The two phases are then emulsified at a high speed with a specialized mixer and the mixing is continued until the solvent in the dispersed phase is evaporated and the particles are hardened or formed. Particles are then collected by filtration, centrifugation, or other method specific for the particle size. For particles with core made of the poly lactic acid or any of its co-polymers with the poly glycolic acid (PLGA), the polymer is typically dissolved in methylene chloride (dispersed phase) and an aqueous solution, with or without a surfactant, is used as the continuous phase (Bodmeier and McGinity, 1987). Depending on the proportion of phases, the target particle size, and the method of particle collection different emulsifiers can be used, including polyvinyl alcohol (PVA), methyl cellulose, polysorbate 80, sodium oleate, and sodium dodecyl sulfate. The type of mixing process, the mixing speed, the concentration of the emulsifier, and the concentration of the core material in the dispersed phase, will together determine the final size and distribution of the resulting particles (Jalil and Nixon, 1990). Rifampicin microparticles for pulmonary delivery were prepared by this method using rifampicin and PLGA dissolved in methylene chloride as the dispersed phase to form the core of the particle and the continuous phase consisted of an aqueous glycerol solution containing PVA as the emulsifier (O'Hara and Hickey, 2000). The two phases were mixed at 5000 rpm for 45 min and the particles collected by filtration. The resulting particles had a volume diameter of 3.45–6.84 μm and were considered appropriate for pulmonary delivery.

The main limitations of the emulsification/solvent evaporation method are the size of the batches that can be manufactured at once and the number of materials that can be employed as core for the particles. In the past decades a number of alternative techniques for the manufacture of particles have emerged and the field has been named “Particle Engineering.” A few of the advantages of engineered particles are scalability of the methods, enhanced physical and chemical stability of particles, better control of the time of drug release, and in many cases improved bioavailability (Van Oort and Sacchetti, 2007). Spray drying in particular has been a popular technique to manufacture respirable particles due to the variety of materials that can be employed and the range of particle sizes and batch sizes that can be produced. Spray drying is a one-step process that produces particles from a liquid feed (Van Oort and Sacchetti, 2007). Typically, the liquid feed is a solution or suspension that is atomized to form a spray that is placed in a contained hot gaseous environment. The spray droplets are dried to form solid particles that are classified by size by means of a cyclone, bag filter, or electrostatic precipitator. The drying temperature, atomization pressure, gas flow, and composition of the liquid feed can be manipulated to produce particles with different characteristics (Garcia-Contreras et al., 2006b). A number of particles containing anti-tubercular agents have been manufactured by spray drying (Table 1), including rifampicin (O'Hara and Hickey, 2000; Suarez et al., 2001a,b), capreomycin (Fiegel et al., 2008), PA-824 (Sung et al., 2009a), para-aminosalicylic acid (Tsapis et al., 2003), and combinations of rifampicin-isoniazid (Sharma et al., 2001), and rifampicin-isoniazide-pyrazinamide (Pandey et al., 2003). Other particle engineering techniques such as spray freeze drying have been developed for heat sensitive molecules (proteins and peptide drugs or vaccines) from variations of the settings and parameters of spray drying (Garmise et al., 2007).

In addition to the enhanced stability, one of the most attractive advantages to formulate vaccines as dry powders for inhalation is that the antigen presenting cells in the lung (macrophages and dendritic cells) tend to uptake particulate forms that are inhaled as part as their house-keeping function. This event can make particles act as adjuvants because depending on their composition and residence time it enhances the immune response greatly in comparison to soluble antigens. Microparticulate systems are known to stimulate the immune response to help Th1 induction (Raychaudhuri and Rock, 1998). Antigens alone or with other adjuvants have been associated or encapsulated in micro- or nano-particles made of lipids, polymers, sugars, aminoacids, or viral vectors. In addition to providing adjuvancy, the encapsulation of antigens into particles has other advantages for pulmonary delivery such as protecting the integrity of antigens, improving the aerosol dispersion of the particles and lung deposition, and in cases even control the time and amount of antigen released from these particles. Despite all these advantages, only a handful of novel vaccines against TB have been formulated into particles for pulmonary administration (Table 2).

**Table 2:** **Novel anti-TB vaccine formulations for pulmonary administration**.

**Vaccine**	**Formulation**	**Results**	**References**
**SUBUNIT VACCINES**			
HLA-A^*^0201-restricted T-cell epitopes derived from *MTB* antigens: 19 kD, Ag85B (2 epitopes), Ag85A, PstA1, ThyA and RpoB, and ESAT-6	Chitosan-DNA nanoparticles	Levels of IFN-gamma after spray instillation of a suspension of these nanoparticles were much higher in transgenic mice compared to those after spray instillation of the nanoparticles alone or the plasmid by IM injection	Bivas-Benita et al., 2004
Ag85 + trehalose dibehenate	PLGA microspheres	PLGArAg85B microspheres induced the specific hybridoma cells to produce IL-2 at a level that was two orders of magnitude larger than the response elicited by soluble rAg85B	Lu et al., 2007
–	–	A strategy of BCG prime-PLGArAg85B aerosol boost appeared to enhance protection from bacterial infection, as indicated by a reduction in CFU in both the lungs and spleens compared with untreated controls	Lu et al., 2010
DNA encoding the MTB latency antigen Rv1733c	PLGA-polyethyleneimine nanoparticles	Rv1733c DNA-PLGA–PEI nanoparticles increased T cell proliferation and IFN-γ production more potently compared to the same vaccinations given IM	Bivas-Benita et al., 2009
**WHOLE INACTIVATED BACTERIA**			
BCG Danish strain	Leucine microparticles	The bacterial burden of lungs of animals immunized by the pulmonary route with BCG particles was significantly lower than that of animals immunized by BCG SC or untreated controls. Histopathological analysis showed very few and small areas affected by granulomas in animals vaccinated by pulmonary route, compared to those vaccinated SC	Garcia-Contreras et al., 2008

## Advances in the prevention and treatment of TB with aerosolized anti-tuberculosis drugs and vaccines

### Capreomycin powder aerosols

Initial studies investigated the disposition of capreomycin in healthy guinea pigs to define initial PK parameters, which will require subsequent evaluation and redefinition in infected animals. PK studies indicated that, 2 h after receiving the dose, capreomycin plasma concentrations in guinea pigs were similar after i.v. and pulmonary administration (Garcia-Contreras et al., 2007). Capreomycin was also cleared at a lower rate when delivered by the pulmonary route than when given i.v. or i.m., resulting in a significantly longer *t*_1/2_. Thus, it was proposed that given the comparably high drug concentrations in the lungs achieved by inhalation relative to those reported for other inhaled TB drugs (Tsapis et al., 2003) and the relatively good bioavailability achieved by inhalation, efficacy might be achieved in an infection model at an inhaled dose below the dose required to achieve efficacy by conventional parenteral routes of delivery. To test this hypothesis, capreomycin was delivered by aerosol and injection to TB-infected guinea pigs daily for four weeks (Garcia-Contreras et al., 2007). The lungs of animals receiving the highest aerosol dose of capreomycin showed significantly smaller degrees of inflammation, bacterial burdens (CFU/ml), and percentages of lung tissue affected by granulomas and caseous necrosis (by histopathology) than those for any other treatment. In contrast, only animals treated by i.m., injection showed a significantly reduced bacterial burden in the spleen. However, histopathological analysis of spleens revealed a positive effect of treatment with the high dose of capreomycin particles and the i.m., solution. The modest effect in the spleen was attributed to the low systemic concentrations of the drug in animals receiving dry powder aerosols and the actual deposited dose of the drug in the alveoli. It was estimated that the actual deposited doses of aerosolized capreomycin were 10-fold smaller because the aerosols to treat TB-infected animals were delivered by passive inhalation, whereas the PK studies were performed delivering capreomycin aerosols by a direct administration bolus (Garcia-Contreras et al., 2007). Therefore, there is reason to believe that deposition of sufficient drug in the lungs of guinea pigs would potentially result in therapeutic systemic concentrations.

### Dry powder PA-824 aerosols

PA-824, typically administered orally, demonstrates great promise in treating both active and latent TB, with the potential of also treating MDR-TB. We postulated that by delivering drug containing powders directly to the lungs, high concentrations could be achieved to treat the bacteria locally, thus minimizing systemic exposure (Garcia-Contreras et al., 2010). PA-824 was formulated into a stable dry powder porous particle form for delivery by the pulmonary route via simple DPIs. This formulation showed to maintain physical, aerodynamic, and chemical stability at room temperature for six months and under refrigerated conditions for more than one year (Sung et al., 2009a). TB infected guinea pigs were treated with the different PA-824 formulations by the oral and pulmonary routes daily for four weeks. The treatment was well tolerated and no adverse effects were observed in any of the animals. Treatment with aerosolized PA-824 particles appeared to reduce manifestations of disease in the lungs and spleens of guinea pigs. Animals receiving low and high doses of inhaled PA-824 aerosols showed significantly less inflammation (as indicated by wet tissue weights), a lower number of viable bacteria, and less tissue damage (as shown by histopathological analysis) than untreated animals or those inhaling placebo particles. Furthermore, bacterial burden was even lower in the spleens of animals that inhaled the high dose of PA-824 particles than in those that inhaled the low dose, and tissue damage was observed to a lesser extent in those animals as well.

### Aerosolized vaccines against TB

The BCG powder vaccine, manufactured by Wong et al. (2007) was administered to guinea pigs by the pulmonary route (Garcia-Contreras et al., 2008) and its protective effect compared to that after the traditional intradermal immunization. Animals were challenged with MTB H37Rv aerosols six weeks after immunization and necropsy performed four weeks after challenge. Bacteriological analysis showed that the bacterial burden in the lungs of all immunized animals was significantly lower than that of unimmunized controls, regardless of the BCG dose or the route of immunization. Most importantly, the bacterial burden of lungs of animals immunized by the pulmonary route with BCG particles was significantly lower than that of animals immunized by the traditional intradermal route. Histopathological analysis supported the bacteriology results, showing few granulomas in the lungs of animals vaccinated by the intradermal route, whereas the lung tissue appeared almost normal in animals immunized by the pulmonary route.

The role of alveolar macrophages as initial host cells for MTB was studied in a vaccine strategy with a subunit antigen. PLGA micro-particles were manufactured in respirable sizes as carriers for recombinant Ag85B (rAg85B) to be delivered by pulmonary route as a novel vaccine against TB (Lu et al., 2007). Continuous release of antigens from PLGA has shown to provide a prolonged immunological response in animals avoiding the need for multiple boosting (Shahin et al., 1995; Men et al., 1996). The ability of these r-Ag85B-PLGA microspheres to deliver antigen to macrophages for subsequent processing and presentation to the specific CD4 was assessed in the T-hybridoma cells DB-1. These cells recognize the Ag85B97-112 epitope presented in the context of MHC class II and secrete IL-2 as the cytokine marker. THP-1 macrophage-like cells exposed with r-Ag85B-PLGA microspheres induced production of IL-2 at a level that was two orders of magnitude larger than the response elicited by soluble rAg85B in DB-1 cells (Lu et al., 2007). These results suggested that dry powder delivery of rAg85B using a microsphere formulation had potential as a vaccine strategy for preventing TB or to be employed as a promising boosting vaccine.

The potential of aerosolized r-Ag85B-PLGA microspheres to protect against TB was evaluated in the guinea pig model employing prime-boost strategies with BCG and r-Ag85B-PLGA microparticles (Lu et al., 2010). The r-Ag85B-PLGA micro-particles were prepared in sizes suitable for inhalation and delivered as aerosolized dry powders to the lungs of guinea pigs in single or multiple doses in homologous and heterologous immunization strategies. Immunized animals were challenged with a low-dose aerosol of *MTB* H37Rv to assess the extent of protection. The heterologous strategy of BCG prime-r-Ag85B-PLGA aerosol boosts appeared to enhance protection from bacterial infection, as indicated by a reduction in the bacterial burden in both the lungs and spleens of immunized animals compared with non-vaccinated animals. Even though the bacterial burdens were not statistically different between the BCG and BCG-BCG groups, histopathological and morphometric analyses of lung and spleen tissue indicated the positive effect of BCG-P-rAg85B immunization by measuring a smaller area of tissue affected and number and size of granulomas observed in the tissues of animals immunized by the pulmonary route. Therefore, it was suggested that direct pulmonary immunization using these particles enhanced the protection afforded by primary immunization with BCG against TB infection in this animal model. Since a large proportion of the population of the world has been immunized with BCG as infants, an aerosol boost may be a promising strategy to enhance and prolong the protective immunity based on BCG-initiated immunity (Lu et al., 2010).

## Conclusions

The advantages of aerosol administration of drugs and vaccines for TB treatments are supported by the different aspects presented in this review. These include: (1) higher drug concentrations at the main site of infection limiting systemic side effects, and (2) enhanced protection with the convential vaccines and possible vaccine strategy using subunit vaccines for immune-compromised patients. These advantages are not only clinical, but also pharmaceutical offering more stable formulations and dosage forms that can be stored without refrigeration for their use in regions of the world where the burden of TB infection is high.

### Conflict of interest statement

The authors declare that the research was conducted in the absence of any commercial or financial relationships that could be construed as a potential conflict of interest.
